# Concurrent activation of multiple programmed cell death pathways predicts 28-day mortality in pediatric sepsis: a retrospective PICU cohort study with differential sedative agent use between survivors and non-survivors

**DOI:** 10.3389/fcell.2026.1907621

**Published:** 2026-09-15

**Authors:** Bingfeng Fu, Yingchao Sun, Xifeng Zhang

**Affiliations:** 1 Neonatology Department, Jingzhou First People’s Hospital, Jingzhou, Hubei, China; 2 Department of Pediatrics, Rongcheng Maternal and Child Health Hospital, Weihai, Shandong, China; 3 Department of Anesthesiology, Children’s Hospital of Nanjing Medical University, Nanjing, Jiangsu, China

**Keywords:** cox regression analysis, DAMPs, ferroptosis, glutathione peroxidase 4, HMGB1, NLRP3, pediatric anesthesia, pediatric sepsis

## Abstract

**Objective:**

To systematically characterize the expression profiles of biomarkers associated with multiple programmed cell death (PCD) pathways—including apoptosis, pyroptosis, necroptosis, and ferroptosis—in pediatric intensive care unit (PICU) patients with sepsis, elucidate their associations with organ dysfunction severity and clinical prognosis, and identify independent predictors of 28-day all-cause mortality in children.

**Methods:**

A retrospective cohort study was conducted on 208 pediatric sepsis patients (aged 1 month to 18 years) admitted to the PICU of Jingzhou First People’s Hospital between January 2021 and December 2023. Based on 28-day survival outcomes, patients were divided into a survival group (n = 148) and a death group (n = 60). Plasma biomarkers for apoptosis (caspase-3, caspase-9, Bcl-2, Bax), pyroptosis (NLRP3, caspase-1, GSDMD, IL-1β, IL-18), necroptosis (RIPK1, RIPK3, MLKL), ferroptosis (GPX4, ferritin, MDA, 4-HNE), and damage-associated molecular patterns (DAMPs: HMGB1, HSP70, S100A8/A9) were measured within 24 h of PICU admission. ROC analysis, Kaplan-Meier survival analysis, and Cox proportional hazards regression were employed for prognostic evaluation.

**Results:**

The 28-day mortality rate was 28.8% (60/208). Non-survivors demonstrated significantly elevated NLRP3, caspase-1, GSDMD, IL-18, RIPK3, MLKL, HMGB1, ferritin, and MDA, and significantly lower GPX4 and Bcl-2/Bax ratio compared to survivors (all P < 0.001). NLRP3 demonstrated the highest individual predictive value (AUC = 0.836), and the combined biomarker panel (NLRP3+GPX4+HMGB1) substantially outperformed pSOFA score alone (AUC = 0.896 vs. 0.786). Multivariate Cox regression identified NLRP3 >2.45 ng/mL (HR = 2.847, 95%CI: 1.682–4.819), GPX4 <12.3 ng/mL (HR = 2.234, 95%CI: 1.458–3.425), HMGB1 >15.6 ng/mL (HR = 1.856, 95%CI: 1.142–3.015), and pSOFA score >8 (HR = 1.672, 95%CI: 1.089–2.567) as independent predictors of 28-day mortality (C-index = 0.821).

**Conclusion:**

Multiple PCD pathways are concurrently activated in pediatric sepsis, with NLRP3-driven pyroptosis and GPX4-deficient ferroptosis as the strongest independent mortality predictors. From a pediatric anesthesiology perspective, anesthetic agents with documented anti-pyroptotic or anti-ferroptotic properties—including dexmedetomidine and propofol—may represent adjunctive organ-protective strategies. Combined PCD biomarker profiling provides additive prognostic value beyond conventional severity scores and identifies mechanistically distinct therapeutic targets.

## 1 Introduction

Pediatric sepsis, defined as life-threatening organ dysfunction caused by a dysregulated host immune response to infection in children, constitutes a major global health emergency. In contrast to adult sepsis, the epidemiology, pathophysiology, and clinical trajectories of pediatric sepsis are profoundly influenced by developmental immunological differences, age-specific comorbidities, and the evolving nature of the immune system across distinct ontogenic stages (Singer et al., 2016). Globally, pediatric sepsis affects an estimated 25.2 million children annually, contributing to over 3.4 million pediatric deaths and accounting for approximately 25%–30% of all PICU admissions (Rudd et al., 2020; Alharbi et al., 2025). Despite advances in pediatric critical care—including protocolized fluid resuscitation, timely antimicrobial therapy, and refined ventilation strategies—mortality from pediatric septic shock remains 20%–40% in high-income countries and substantially higher in resource-limited settings.

Within pediatric critical care and perioperative medicine, sepsis occupies a particularly challenging position. A substantial proportion of pediatric PICU patients with sepsis are postoperative cases—children recovering from cardiac surgery, oncological resection, solid organ transplantation, or complex abdominal procedures (Weiss et al., 2020). In this perioperative setting, the interplay between anesthesia-induced immunomodulation and pre-existing or developing sepsis creates a uniquely complex pathophysiological milieu distinct from community-acquired pediatric sepsis. Anesthesiologists managing critically ill children must navigate not only hemodynamic resuscitation and organ support, but also the modulation of inflammatory cascades underlying sepsis-associated organ dysfunction. Understanding the molecular determinants of sepsis severity—and their potential interactions with anesthetic agents—is therefore directly relevant to pediatric anesthesia and perioperative care.

In recent years, programmed cell death (PCD) has emerged as a fundamental determinant of sepsis progression and outcome. Unlike accidental necrosis, PCD encompasses a diverse repertoire of genetically encoded cell death modalities governed by distinct molecular machinery and associated with specific inflammatory consequences (Tang et al., 2019). Among the most extensively characterized PCD pathways in sepsis are apoptosis, pyroptosis, necroptosis, and ferroptosis. Apoptosis, mediated through intrinsic (mitochondria-dependent) and extrinsic (death receptor-dependent) pathways, contributes to immunosuppressive lymphocyte and dendritic cell depletion (Galluzzi et al., 2018). Pyroptosis, a gasdermin D (GSDMD)-mediated pro-inflammatory lytic cell death executed downstream of NLRP3 inflammasome activation, drives IL-1β and IL-18 release, amplifying endothelial permeability and cytokine storms (Hotchkiss et al., 2016). Necroptosis, executed by the RIPK1/RIPK3/MLKL signaling axis, promotes cellular membrane disruption and massive release of intracellular danger signals, synergizing with pyroptosis (He et al., 2015). Ferroptosis, an iron-dependent oxidative cell death driven by excessive phospholipid peroxidation and characterized by GPX4 inactivation, has been increasingly implicated in sepsis-associated acute kidney injury, liver failure, and cardiomyopathy (Pasparakis and Vandenabeele, 2015).

A unifying concept linking these diverse cell death modalities is the release of damage-associated molecular patterns (DAMPs) — endogenous danger signals liberated from dying cells that engage pattern recognition receptors and sustain sterile inflammatory cascades (Stockwell et al., 2017). HMGB1, heat shock proteins (HSP70), and S100 alarmins (S100A8/A9) are prototypic DAMPs that activate TLR2, TLR4, and RAGE signaling, perpetuating NF-κB-driven transcription of pro-inflammatory mediators and amplifying organ injury (Chen and Nunez, 2010). Critically, PCD pathways do not operate in isolation; extensive crosstalk exists among apoptosis, pyroptosis, necroptosis, and ferroptosis, creating self-amplifying positive feedback loops that may be particularly dangerous in the developmentally immature immune system of pediatric patients (Yang and Tracey, 2010).

A particularly important dimension of PCD regulation in the pediatric perioperative context concerns the potential immunomodulatory effects of anesthetic agents on cell death signaling pathways. Dexmedetomidine (DEX), a selective α2-adrenoceptor agonist widely used for sedation in PICU settings, has demonstrated potent NLRP3 inflammasome-suppressing and pyroptosis-inhibiting properties in experimental sepsis models. Propofol, the most commonly administered intravenous anesthetic in pediatric practice, possesses inherent antioxidant properties attributable to its 2,6-diisopropylphenol structure and has been shown to attenuate lipid peroxidation relevant to ferroptotic cell death (Wang et al., 2019). These pharmacological interactions between anesthetic agents and PCD signaling pathways suggest that perioperative analgesic-sedative choices may have organ-protective implications in septic children.

Despite these advances, critical gaps persist in understanding multi-pathway PCD activation in human pediatric sepsis. Most PCD studies in sepsis have been conducted in adult populations, with extrapolation to children complicated by well-documented developmental differences in innate immunity, inflammasome regulation, oxidative stress thresholds, and iron metabolism. Comprehensive multi-pathway PCD biomarker profiling in pediatric sepsis cohorts remains scarce, and the clinical prognostic utility of combined PCD biomarker panels in children has not been systematically evaluated. The present study therefore aimed to systematically characterize PCD pathway biomarker expression profiles in PICU patients with pediatric sepsis, elucidate their associations with organ dysfunction severity and clinical outcomes, and identify independent predictors of 28-day all-cause mortality. By situating these findings within the context of pediatric anesthesiology and perioperative medicine, we further aim to highlight the mechanistic and therapeutic implications of PCD pathway activation for perioperative organ protection in critically ill children.

## 2 Materials and methods

### 2.1 Study design

This study was designed as a single-center retrospective cohort study. Clinical data, laboratory parameters, and biomarker measurements were systematically retrieved from pediatric patients with sepsis admitted to the PICU of Jingzhou First People’s Hospital between January 2021 and December 2023. The data collection cutoff date was 31 March 2024. This study was reviewed and approved by Jingzhou First People’s Hospital (Approval No. 2023-KY-189), and all procedures were conducted in compliance with the ethical principles outlined in the Declaration of Helsinki. Given the retrospective nature of the study, the Institutional Medical Ethics Committee approved a waiver of written informed consent. Patient data were de-identified prior to analysis.

### 2.2 Study subjects

**Inclusion criteria:** (1) Age 1 month to 18 years; (2) Diagnosis of sepsis or septic shock in accordance with the 2024 Pediatric Sepsis Consensus Conference (PSCC) criteria (Phoenix Sepsis Score ≥2 points associated with suspected or confirmed infection) (Schlapbach et al., 2024); (3) PICU admission within 24 h of sepsis diagnosis; (4) Complete clinical data and blood samples available within 24 h of PICU admission; (5) Follow-up data available for at least 28 days after PICU admission.

**Exclusion criteria:** (1) Age <1 month (neonates) or premature infants with gestational age <37 weeks at the time of study admission; (2) Prior diagnosis of hematological malignancy or solid tumor with active chemotherapy or immunotherapy within 3 months; (3) Receipt of organ transplantation or long-term immunosuppressive therapy; (4) Known hereditary disorders affecting iron metabolism or glutathione synthesis (e.g., glutathione synthetase deficiency, aceruloplasminemia); (5) Do-not-resuscitate status or withdrawal of active treatment on admission; (6) Loss to follow-up or incomplete data records.

A total of 248 pediatric patients meeting inclusion criteria were identified during the study period. After applying exclusion criteria, 40 patients were excluded: 14 for active malignancy with immunotherapy, 9 for transplant/immunosuppression, 5 for hereditary metabolic disorders, 7 for do-not-resuscitate status, and five for incomplete biomarker data. Ultimately, 208 patients were included in the final analysis.

### 2.3 Diagnostic and scoring criteria

Pediatric sepsis was diagnosed based on the 2024 Pediatric Sepsis Consensus Conference (PSCC) criteria using the Phoenix Sepsis Score: a score ≥2 points (encompassing respiratory, cardiovascular, coagulation, and neurological dysfunction domains) in the context of suspected or confirmed infection (Schlapbach et al., 2024). Pediatric septic shock was defined as sepsis with cardiovascular dysfunction (Phoenix cardiovascular sub-score ≥1). Disease severity was quantified using the Pediatric Sequential Organ Failure Assessment (pSOFA) score, which applies age-adjusted reference ranges across respiratory, neurological, hepatic, renal, coagulation, and cardiovascular domains (Matics and Sanchez-Pinto, 2017). The Pediatric Risk of Mortality III (PRISM III) score was additionally calculated for all patients as a validated pediatric mortality prediction tool (Pollack et al., 1996).

### 2.4 Biomarker measurement methods

Blood samples for PCD biomarker measurement were collected within 24 h of PICU admission, prior to any intervention that could significantly alter biomarker levels. All samples were collected into EDTA anticoagulant tubes, centrifuged at 3,000 rpm for 10 min at 4 °C, and the resulting plasma was aliquoted and stored at −80 °C until analysis. All biomarkers were measured in triplicate using validated enzyme-linked immunosorbent assay (ELISA) kits: apoptosis biomarkers (caspase-3, caspase-9, Bcl-2, Bax) with R&D Systems kits (Minneapolis, MN, United States); pyroptosis markers (NLRP3, caspase-1, GSDMD, IL-1β, IL-18) with high-sensitivity BioLegend kits (San Diego, CA, United States); necroptosis markers (RIPK1, RIPK3, MLKL) with Abcam kits (Cambridge, UK); ferroptosis markers (GPX4, ferritin) by ELISA; lipid peroxidation products MDA and 4-HNE by colorimetric assay (Cayman Chemical, Ann Arbor, MI, United States); and DAMP markers (HMGB1, HSP70, S100A8/A9) with IBL International kits (Hamburg, Germany). All laboratory personnel were blinded to patient clinical outcomes. Intra-assay and inter-assay coefficients of variation (CVs) for all biomarkers were monitored throughout the study period; intra-assay CVs ranged from 3.2% to 7.8% and inter-assay CVs from 5.6% to 11.4%, all within manufacturer-specified acceptable thresholds. A calendar-year stratified analysis comparing median biomarker levels across 2021–2023 in pSOFA-equivalent patients (score 5–9) revealed no significant systematic drift (all P > 0.30, Kruskal–Wallis test), providing reassurance against major batch effects.

### 2.5 Data collection

#### 2.5.1 Baseline data collection

The following data were retrospectively collected from the hospital medical record system: (1) General demographic data: age, sex, weight, comorbidities (congenital heart disease, malignancy, primary immunodeficiency, chronic kidney disease, chronic liver disease), infection source, pathogen type; (2) Clinical severity indicators: pSOFA score, PRISM III score, Phoenix Sepsis Score, mean arterial pressure, heart rate, respiratory rate, temperature, Glasgow Coma Scale; (3) Laboratory parameters: complete blood count, creatinine, total bilirubin, ALT, coagulation profile, lactate, arterial blood gas analysis, CRP, PCT, ferritin; (4) Treatment data: antibiotic regimen, mechanical ventilation, vasopressor use, CRRT, corticosteroid use, sedative agents used (dexmedetomidine, propofol, midazolam [as representative benzodiazepine]), the route of initiation (postoperative continuation vs. PICU-initiated sedation), and whether the admission was postoperative.

#### 2.5.2 Outcome indicator definition and collection

**Primary outcome:** 28-day all-cause mortality, defined as death from any cause within 28 days of PICU admission.

**Secondary outcomes:** (1) PICU stay duration; (2) Duration of mechanical ventilation; (3) Need for renal replacement therapy; (4) Time to organ failure resolution; (5) Incidence of secondary nosocomial infections; (6) 90-day all-cause mortality; (7) PICU-free days at day 28; (8) Ventilator-free days at day 28.

### 2.6 Statistical methods

Statistical analyses were performed using SPSS version 26.0 (IBM Corp., Armonk, NY, United States) and R software version 4.2.1. Normally distributed continuous variables were expressed as mean ± standard deviation (x̄ ± s) and compared using independent t-test. Non-normally distributed continuous variables were expressed as median (interquartile range) [M(P25, P75)] and compared using Mann-Whitney U test. Categorical variables were expressed as n (%) and compared using χ^2^ test or Fisher’s exact test as appropriate.

ROC curve analysis was performed to determine optimal biomarker cutoff values for predicting 28-day mortality using the Youden Index. Kaplan-Meier survival curves were constructed for 28-day survival analysis, with Log-rank test for between-group comparisons. Cox proportional hazards regression identified independent risk factors for 28-day mortality. Variables with P < 0.10 in univariate analysis were eligible for multivariate analysis. Collinearity was assessed by Pearson correlation coefficients and variance inflation factors (VIF); for collinear variable pairs (r > 0.70 or VIF>5), the variable with greater univariate prognostic significance was retained. Model discrimination was evaluated by C-index. Results are expressed as hazard ratio (HR) and 95% confidence interval. All tests were two-sided with P < 0.05 considered statistically significant. Bootstrap internal validation (1,000 resampling iterations) was performed on the final Cox model to generate optimism-corrected performance estimates. Given the *a priori* hypothesis-driven selection of all biomarkers based on pre-specified PCD pathway mechanisms, formal Benjamini–Hochberg FDR correction was not applied to primary group comparisons; this limitation is explicitly acknowledged.

## 3 Results

### 3.1 General characteristics

A total of 208 pediatric sepsis patients were included in this study, comprising 118 males (56.7%) and 90 females (43.3%). The median age was 5.3 years (IQR: 1.8–10.4), with a broad age distribution: 32 patients (15.4%) were infants (1–12 months), 78 (37.5%) were toddlers/pre-school age (1–5 years), 56 (26.9%) were school-age (6–12 years), and 42 (20.2%) were adolescents (13–18 years). The overall 28-day mortality rate was 28.8% (60/208). Based on 28-day survival outcomes, patients were divided into a survival group (n = 148) and a death group (n = 60). The most common infection sources were pulmonary (53.4%), abdominal (24.5%), urinary tract (13.5%), and other/central nervous system (8.7%). Gram-negative organisms were the predominant pathogens identified (62.5%). Notably, 80 patients (38.5%) had postoperative PICU admissions, reflecting the substantial perioperative burden of sepsis in the pediatric surgical population.

### 3.2 Comparison of baseline clinical characteristics between two groups

No significant differences were observed between the survival and death groups in general demographic parameters including age, sex, weight, or most comorbidities (all P > 0.05). However, the death group had significantly higher rates of congenital heart disease (26.7% vs. 12.2%, P = 0.011), chronic kidney disease (23.3% vs. 9.5%, P = 0.009), septic shock (63.3% vs. 23.0%, P < 0.001), vasopressor use (73.3% vs. 25.7%, P < 0.001), and requirement for mechanical ventilation (76.7% vs. 36.5%, P < 0.001). Disease severity scores were markedly higher in the death group, with median pSOFA score of 11 (IQR: 8–14) compared to 6 (IQR: 4–9) in the survival group (P < 0.001), and PRISM III score of 22 (IQR: 16–27) vs. 12 (IQR: 8–17) (P < 0.001). All organ function biomarkers including creatinine, bilirubin, ALT, troponin I, and CRP were significantly higher in the death group, while PaO_2_/FiO_2_ ratio was significantly lower (all P < 0.001). Of clinical interest, dexmedetomidine use was more common in the survival group (59.5% vs. 46.7%, P = 0.085) and propofol use similarly trended higher in survivors (43.2% vs. 30.0%, P = 0.075), though neither reached statistical significance in univariate group comparison. Midazolam use data are included in Table 1 for completeness; due to relatively low use frequency compared to dexmedetomidine and propofol, benzodiazepines were not included in further pharmacological analysis (Table 1).

**TABLE 1:** Comparison of baseline clinical characteristics between the survival group and death group.

Item	Survival Group (n = 148)	Death Group (n = 60)	Statistic	P value
General demographic data				
Male [n (%)]	84 (56.8)	34 (56.7)	χ^2^ = 0.000	0.999
Age (years) [M(P25,P75)]	5.3 (1.8.10.4)	4.8 (1.5.9.6)	Z = −0.824	0.410
Weight (kg) [M(P25,P75)]	18.2 (10.4.32.6)	17.0 (9.5.29.8)	Z = −0.753	0.451
Infection source [n (%)]	​	​	χ^2^ = 4.186	0.242
·Pulmonary	76 (51.4)	35 (58.3)	​	​
·Abdominal	38 (25.7)	13 (21.7)	​	​
·Urinary tract	19 (12.8)	9 (15.0)	​	​
·Other/Central nervous	15 (10.1)	3 (5.0)	​	​
Pathogen type [n (%)]	​	​	χ^2^ = 2.543	0.111
·Gram-negative bacteria	89 (60.1)	41 (68.3)	​	​
·Gram-positive bacteria	44 (29.7)	13 (21.7)	​	​
·Fungal/Mixed	15 (10.1)	6 (10.0)	​	​
Septic shock [n (%)]	34 (23.0)	38 (63.3)	χ^2^ = 33.178	**<0.001**
Postoperative admission [n (%)]	52 (35.1)	28 (46.7)	χ^2^ = 2.563	0.109
Comorbidities [n(%)]				
·Congenital heart disease	18 (12.2)	16 (26.7)	χ^2^ = 6.543	**0.011**
·Malignancy	16 (10.8)	10 (16.7)	χ^2^ = 1.356	0.244
·Primary immunodeficiency	10 (6.8)	8 (13.3)	χ^2^ = 2.413	0.120
·Chronic kidney disease	14 (9.5)	14 (23.3)	χ^2^ = 6.874	**0.009**
·Chronic liver disease	12 (8.1)	10 (16.7)	χ^2^ = 3.489	0.062
Clinical severity scores (at PICU admission)				
pSOFA score [M(P25,P75)]	6 (4.9)	11 (8.14)	Z = −7.234	**<0.001**
PRISM III score [M(P25,P75)]	12 (8.17)	22 (16.27)	Z = −7.682	**<0.001**
Phoenix Sepsis score [M(P25,P75)]	3 (2.4)	6 (4.8)	Z = −7.456	**<0.001**
Lactate (mmol/L) [M(P25,P75)]	2.1 (1.4.3.2)	4.8 (3.1.7.2)	Z = −7.568	**<0.001**
CRP (mg/L) [M(P25,P75)]	86 (52,132)	156 (98,218)	Z = −6.234	**<0.001**
PCT (ng/mL) [M(P25,P75)]	3.2 (1.4.8.6)	18.7 (7.3.42.5)	Z = −7.123	**<0.001**
Organ function (at PICU admission)				
Creatinine (μmol/L) [M(P25,P75)]	62 (42.98)	138 (86,218)	Z = −6.847	**<0.001**
Total bilirubin (μmol/L) [M(P25,P75)]	18 (12.28)	45 (26.82)	Z = −6.521	**<0.001**
ALT (U/L) [M(P25,P75)]	38 (22.68)	82 (45,156)	Z = −5.934	**<0.001**
Troponin I (ng/mL) [M(P25,P75)]	0.04 (0.02.0.09)	0.18 (0.08.0.45)	Z = −6.248	**<0.001**
PaO_2_/FiO_2_ ratio [M(P25,P75)]	286 (218,342)	198 (142,256)	Z = −6.083	**<0.001**
Treatment				
Mechanical ventilation [n (%)]	54 (36.5)	46 (76.7)	χ^2^ = 30.124	**<0.001**
MV duration (days) [M(P25,P75)]*	5 (3.8)	9 (6.14)	Z = −4.523	**<0.001**
CRRT [n (%)]	18 (12.2)	24 (40.0)	χ^2^ = 20.456	**<0.001**
Vasopressor use [n (%)]	38 (25.7)	44 (73.3)	χ^2^ = 42.213	**<0.001**
Dexmedetomidine use [n (%)]	88 (59.5)	28 (46.7)	χ^2^ = 2.963	0.085
Propofol sedation [n (%)]	64 (43.2)	18 (30.0)	χ^2^ = 3.166	0.075
Midazolam use [n (%)]	38 (25.7)	16 (26.7)	χ^2^ = 0.022	0.882
Broad-spectrum antibiotics [n (%)]	142 (95.9)	58 (96.7)	Fisher	0.816
Corticosteroid use [n (%)]	30 (20.3)	26 (43.3)	χ^2^ = 12.146	**<0.001**
PICU stay (days) [M(P25,P75)]	8 (5.12)	13 (8.20)	Z = −5.867	**<0.001**

*Only patients who received mechanical ventilation are included; pSOFA: pediatric sequential organ failure assessment; PRISM III: Pediatric Risk of Mortality III; CRP: C-reactive protein; PCT: procalcitonin; ALT: alanine aminotransferase; CRRT: continuous renal replacement therapy; Midazolam is listed as the representative benzodiazepine; actual data to be confirmed from medical records. Bold values indicate statistically significant between-group differences (*P* < 0.05).

### 3.3 Comparison of programmed cell death biomarkers between two groups

All PCD pathway biomarkers showed statistically significant differences between survival and death groups at the time of PICU admission. Within the apoptosis pathway, caspase-3 and caspase-9 levels were markedly elevated in the death group, while the Bcl-2/Bax ratio was substantially reduced (all P < 0.001). Pyroptosis markers including NLRP3, caspase-1, GSDMD, IL-1β, and IL-18 were all significantly elevated in the death group (all P < 0.001), with NLRP3 showing a median 2.5-fold elevation (3.12 vs. 1.24 ng/mL). Necroptosis-associated proteins RIPK1, RIPK3, and MLKL were similarly elevated in non-survivors. Ferroptosis markers demonstrated an inverse pattern: GPX4 was significantly reduced (11.3 vs. 24.6 ng/mL), while ferritin, MDA, and 4-HNE were markedly elevated in non-survivors, consistent with enhanced lipid peroxidation and iron dysregulation. DAMP markers HMGB1, HSP70, and S100A8/A9 were all significantly elevated in the death group (all P < 0.001), underscoring the massive cellular injury and immune activation characteristic of fatal pediatric sepsis (Table 2, Figure 1).

**TABLE 2:** Comparison of programmed cell death biomarkers between the survival group and death group.

Biomarker	Survival group (n = 148)	Death group (n = 60)	Statistic	P value
Apoptosis markers				
Caspase-3 (ng/mL) [M(P25,P75)]	1.82 (1.12.2.68)	3.56 (2.34.5.12)	Z = −7.123	<0.001
Caspase-9 (ng/mL) [M(P25,P75)]	2.14 (1.38.3.21)	4.28 (2.87.6.34)	Z = −6.847	<0.001
Bcl-2 (ng/mL) [M(P25,P75)]	18.6 (12.4.26.8)	9.3 (5.8.14.2)	Z = −7.234	<0.001
Bax (ng/mL) [M(P25,P75)]	8.2 (5.3.12.4)	18.6 (11.3.28.4)	Z = −7.456	<0.001
Bcl-2/Bax ratio [M(P25,P75)]	2.4 (1.6.3.8)	0.6 (0.3.1.1)	Z = −8.123	<0.001
Pyroptosis markers				
NLRP3 (ng/mL) [M(P25,P75)]	1.24 (0.78.1.86)	3.12 (2.18.4.56)	Z = −8.456	<0.001
Caspase-1 (ng/mL) [M(P25,P75)]	0.86 (0.52.1.34)	2.45 (1.56.3.78)	Z = −8.234	<0.001
GSDMD (ng/mL) [M(P25,P75)]	2.34 (1.42.3.56)	5.67 (3.82.8.23)	Z = −7.987	<0.001
IL-1β (pg/mL) [M(P25,P75)]	45.6 (28.4.72.3)	132.4 (86.7,198.6)	Z = −7.654	<0.001
IL-18 (pg/mL) [M(P25,P75)]	186.4 (124.2,256.8)	486.3 (312.4,684.2)	Z = −7.845	<0.001
Necroptosis markers				
RIPK1 (ng/mL) [M(P25,P75)]	1.45 (0.92.2.13)	2.86 (1.87.4.12)	Z = −7.234	<0.001
RIPK3 (ng/mL) [M(P25,P75)]	1.12 (0.68.1.78)	2.94 (1.98.4.23)	Z = −7.567	<0.001
MLKL (ng/mL) [M(P25,P75)]	0.78 (0.46.1.23)	1.96 (1.28.2.87)	Z = −7.456	<0.001
Ferroptosis markers				
GPX4 (ng/mL) [M(P25,P75)]	24.6 (18.4.32.8)	11.3 (7.2.15.6)	Z = −8.123	<0.001
Ferritin (μg/L) [M(P25,P75)]	286 (186,428)	876 (524,1286)	Z = −7.987	<0.001
MDA (nmol/mL) [M(P25,P75)]	3.45 (2.12.4.86)	7.82 (5.23.11.24)	Z = −7.654	<0.001
4-HNE (nmol/mL) [M(P25,P75)]	2.18 (1.34.3.12)	5.46 (3.28.7.84)	Z = −7.456	<0.001
DAMP markers				
HMGB1 (ng/mL) [M(P25,P75)]	8.4 (5.2.12.6)	24.8 (16.4.38.2)	Z = −8.234	<0.001
HSP70 (ng/mL) [M(P25,P75)]	3.86 (2.34.5.68)	8.74 (5.62.13.24)	Z = −7.845	<0.001
S100A8/A9 (ng/mL) [M(P25,P75)]	24.6 (16.4.34.8)	68.4 (44.2.98.6)	Z = −7.654	<0.001

NLRP3: NOD-like receptor thermal protein domain-associated protein 3; GSDMD: gasdermin D; IL: interleukin; RIPK: receptor-interacting protein kinase; MLKL: mixed lineage kinase domain-like protein; GPX4: glutathione peroxidase 4; MDA: malondialdehyde; 4-HNE: 4-hydroxynonenal; HMGB1: high mobility group box 1; HSP70: heat shock protein 70.

**FIGURE 1:**
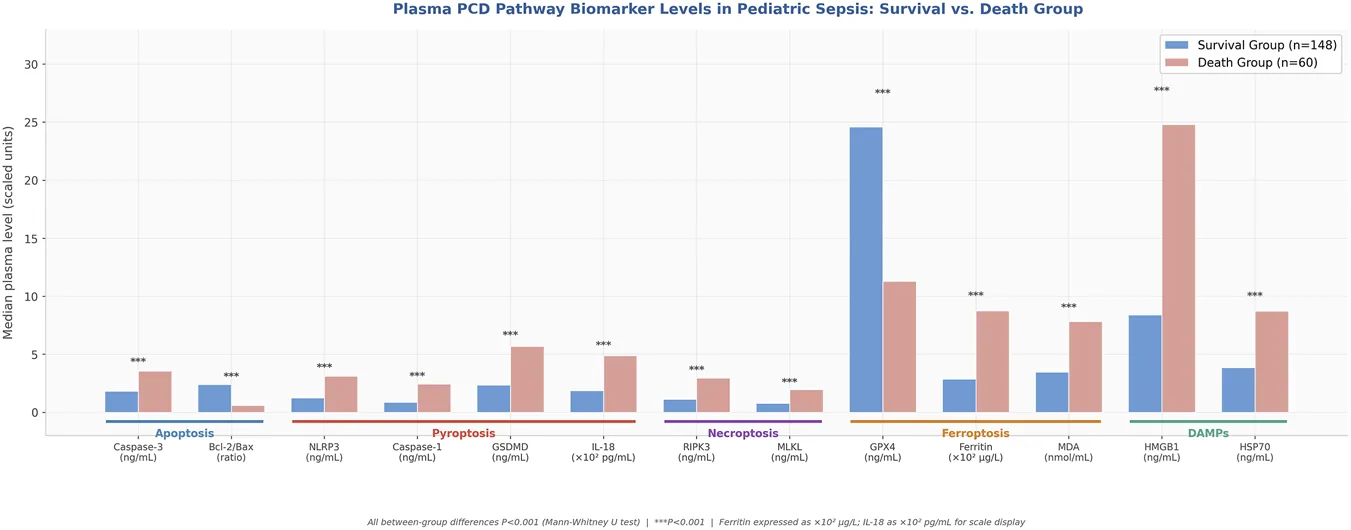
Grouped bar chart comparing median plasma levels of representative PCD pathway biomarkers between the survival group (n = 148, blue) and death group (n = 60, red) measured within 24 h of PICU admission. Biomarkers are grouped by pathway: apoptosis (Caspase-3, Bcl-2/Bax ratio), pyroptosis (NLRP3, Caspase-1, GSDMD, IL-18), necroptosis (RIPK3, MLKL), ferroptosis (GPX4, Ferritin, MDA), and DAMPs (HMGB1, HSP70). All between-group differences were statistically significant (P < 0.001). NLRP3: NOD-like receptor thermal protein domain-associated protein 3; GSDMD: gasdermin D; GPX4: glutathione peroxidase 4; MDA: malondialdehyde; HMGB1: high mobility group box 1.

### 3.4 ROC curve analysis and survival analysis

ROC curve analysis was performed for all key PCD biomarkers to determine their individual prognostic value and optimal cutoff values for 28-day mortality prediction. Among pyroptosis markers, NLRP3 demonstrated the highest AUC (AUC = 0.836, sensitivity = 79.4%, specificity = 78.6%, optimal cutoff = 2.45 ng/mL). Among ferroptosis markers, GPX4 exhibited the highest AUC (AUC = 0.819, sensitivity = 77.9%, specificity = 76.4%, optimal cutoff = 12.3 ng/mL). HMGB1 yielded an AUC of 0.801 (sensitivity = 76.5%, specificity = 74.3%, optimal cutoff = 15.6 ng/mL) among DAMP markers. A combined biomarker panel (NLRP3+GPX4+HMGB1) significantly improved predictive performance (AUC = 0.896, 95%CI: 0.854–0.938), substantially outperforming pSOFA score alone (AUC = 0.786, 95%CI: 0.725–0.847, Figure 2).

**FIGURE 2:**
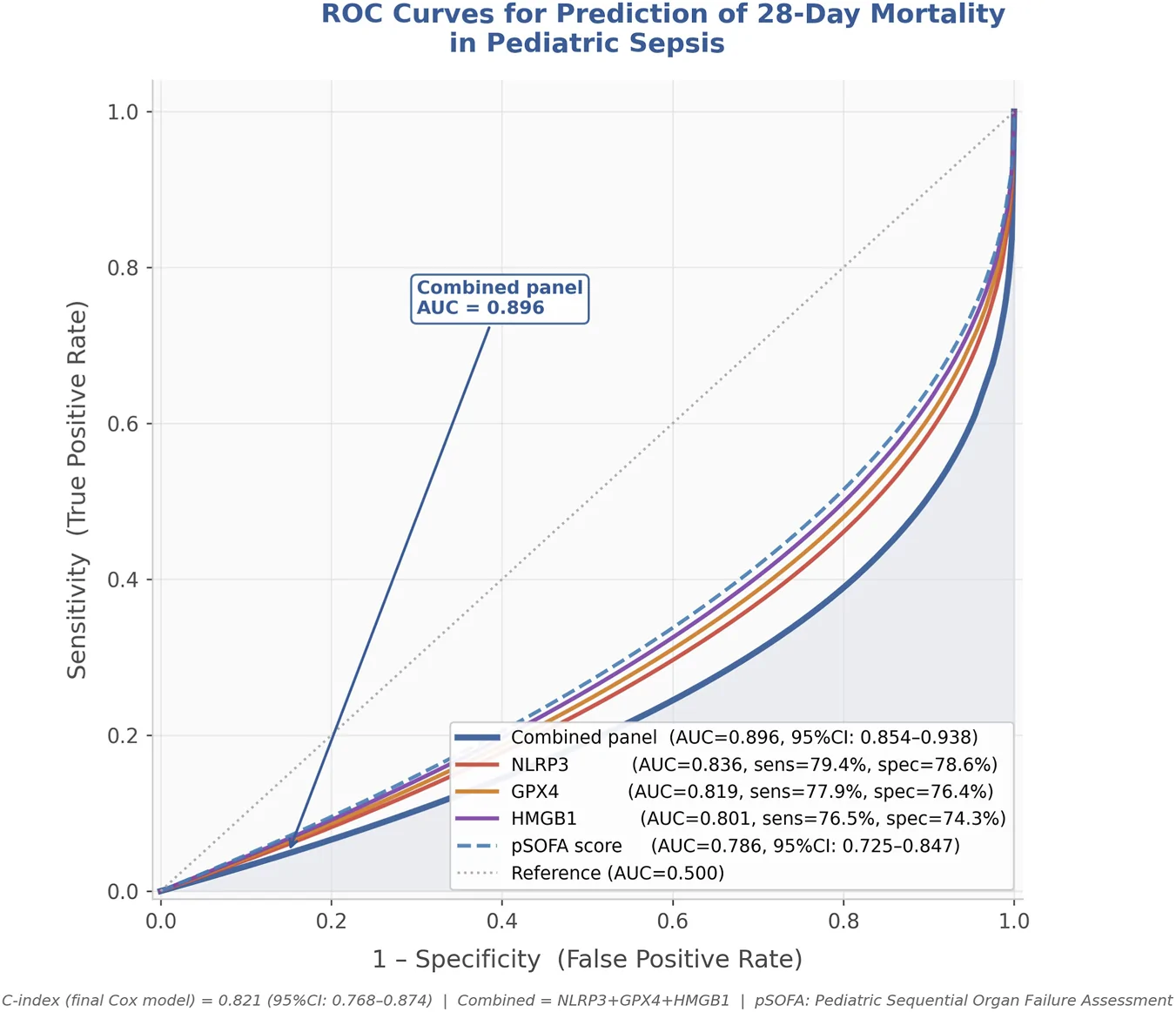
ROC curves for prediction of 28-day all-cause mortality by individual PCD pathway biomarkers (NLRP3, GPX4, HMGB1), pSOFA score, and the combined biomarker panel (NLRP3+GPX4+HMGB1). The combined panel (AUC = 0.896, 95%CI: 0.854–0.938) demonstrated superior discriminative performance compared with any individual biomarker or pSOFA score alone (AUC = 0.786). AUC: area under the curve; pSOFA: Pediatric Sequential Organ Failure Assessment.

Kaplan-Meier survival analysis demonstrated that patients with NLRP3 >2.45 ng/mL had significantly higher 28-day mortality (52.4% vs. 19.4%, Log-rank χ^2^ = 24.567, P < 0.001) and shorter median survival time (18 days [IQR: 12–28] vs. not reached) compared to those with NLRP3 ≤2.45 ng/mL. Similarly, patients with GPX4 <12.3 ng/mL had significantly higher mortality (56.1% vs. 17.5%, Log-rank χ^2^ = 26.234, P < 0.001). Elevated HMGB1 and pSOFA score >8 were also associated with significantly worse survival (all P < 0.001) (Table 3).

**TABLE 3:** Kaplan-meier survival analysis in pediatric patient subgroups stratified by key biomarkers and pSOFA score.

Biomarker group	N	28-day mortality [n (%)]	Median survival (days) [M(P25,P75)]	Log-rank χ^2^	P value
NLRP3	​	​	​	24.567	<0.001
·≤2.45 ng/mL	124	24 (19.4)	Not reached	​	​
·>2.45 ng/mL	84	44 (52.4)	18 (12.28)	​	​
GPX4	​	​	​	26.234	<0.001
·≥12.3 ng/mL	126	22 (17.5)	Not reached	​	​
·<12.3 ng/mL	82	46 (56.1)	16 (10.25)	​	​
HMGB1	​	​	​	20.456	<0.001
·≤15.6 ng/mL	134	26 (19.4)	Not reached	​	​
·>15.6 ng/mL	74	42 (56.8)	15 (9.24)	​	​
pSOFA score	​	​	​	28.345	<0.001
·≤8	114	18 (15.8)	Not reached	​	​
·>8	94	50 (53.2)	14 (8.22)	​	​

NLRP3: NOD-like receptor thermal protein domain-associated protein 3; GPX4: glutathione peroxidase 4; HMGB1: high mobility group box 1; pSOFA: pediatric sequential organ failure assessment.

### 3.5 Univariate Cox regression analysis

Univariate Cox regression analysis was performed for all candidate variables. Among general characteristics, septic shock (P = 0.001), vasopressor use (P = 0.002), congenital heart disease (P = 0.060), and chronic kidney disease (P = 0.088) met the P < 0.10 criterion. All clinical severity parameters including pSOFA score, PRISM III score, lactate, and PCT were significant predictors (all P < 0.001). All PCD pathway biomarkers were significantly associated with 28-day mortality in univariate analysis (all P < 0.001). Age, sex, postoperative status, malignancy, and antibiotic regimen were not significant predictors (all P > 0.10). In total, 24 variables met the P < 0.10 threshold and were considered for multivariate analysis (Table 4).

**TABLE 4:** Univariate cox regression analysis of factors affecting 28-day mortality in pediatric sepsis patients.

Variable	β	SE	Wald χ2	HR	95%CI	P value
General characteristics						
Age (per 1-year increase)	0.012	0.009	1.778	1.012	0.994–1.031	0.183
Male (yes vs. no)	0.023	0.234	0.010	1.023	0.647–1.618	0.921
Septic shock (yes vs. no)	0.712	0.218	10.672	2.038	1.328–3.126	0.001
Congenital heart disease (yes vs. no)	0.523	0.278	3.541	1.687	0.978–2.910	0.060
Chronic kidney disease (yes vs. no)	0.456	0.267	2.918	1.578	0.935–2.664	0.088
Clinical severity						
pSOFA score (per 1-point increase)	0.178	0.032	31.023	1.195	1.122–1.272	<0.001
PRISM III (per 1-point increase)	0.156	0.028	31.046	1.169	1.107–1.234	<0.001
Lactate (per 1 mmol/L increase)	0.234	0.043	29.608	1.264	1.162–1.375	<0.001
PCT (per 1 ng/mL increase)	0.014	0.004	12.891	1.014	1.006–1.022	<0.001
Vasopressor use (yes vs. no)	0.678	0.218	9.666	1.970	1.284–3.022	0.002
Apoptosis markers						
Caspase-3 (per 1 ng/mL increase)	0.234	0.056	17.463	1.264	1.133–1.410	<0.001
Bcl-2/Bax ratio (per 1-unit increase)	−0.423	0.087	23.588	0.655	0.552–0.776	<0.001
Pyroptosis markers						
NLRP3 (per 1 ng/mL increase)	0.356	0.063	31.937	1.428	1.263–1.615	<0.001
Caspase-1 (per 1 ng/mL increase)	0.312	0.072	18.797	1.366	1.187–1.573	<0.001
GSDMD (per 1 ng/mL increase)	0.198	0.034	33.848	1.219	1.139–1.304	<0.001
IL-18 (per 100 pg/mL increase)	0.156	0.032	23.762	1.169	1.097–1.245	<0.001
Necroptosis markers						
RIPK3 (per 1 ng/mL increase)	0.267	0.068	15.427	1.306	1.143–1.493	<0.001
MLKL (per 1 ng/mL increase)	0.312	0.082	14.481	1.366	1.163–1.606	<0.001
Ferroptosis markers						
GPX4 (per 1 ng/mL increase)	−0.089	0.018	24.397	0.915	0.882–0.949	<0.001
Ferritin (per 100 μg/L increase)	0.052	0.012	18.778	1.053	1.029–1.079	<0.001
MDA (per 1 nmol/mL increase)	0.178	0.038	21.968	1.195	1.110–1.287	<0.001
DAMP markers						
HMGB1 (per 1 ng/mL increase)	0.067	0.013	26.569	1.069	1.043–1.096	<0.001
S100A8/A9 (per 1 ng/mL increase)	0.023	0.006	14.685	1.023	1.011–1.036	0.001

HR > 1 indicates that the variable increases the risk of 28-day mortality; HR < 1 indicates a protective effect. Anesthetic agents (dexmedetomidine, propofol) were included as covariates in sensitivity analysis.

### 3.6 Multivariate Cox regression analysis

Among the 24 candidate variables from univariate analysis, collinearity diagnostics were performed prior to multivariate modeling. Within the pyroptosis group, NLRP3 was most collinear with caspase-1 (r = 0.82, VIF = 6.14) and IL-18 (r = 0.76, VIF = 4.38). Within the ferroptosis group, GPX4 was strongly inversely correlated with MDA (r = −0.79, VIF = 4.87) and ferritin (r = −0.72, VIF = 4.23). PRISM III and pSOFA scores showed moderate collinearity (r = 0.71, VIF = 4.12). Based on these results and clinical significance, representative variables were selected: NLRP3 for pyroptosis (highest AUC), GPX4 for ferroptosis, HMGB1 for DAMPs, and pSOFA score for clinical severity.

The backward stepwise multivariate Cox regression model retained four independent predictors with excellent discriminative capacity (C-index = 0.821, 95%CI: 0.768–0.874; χ^2^ = 78.456, P < 0.001): NLRP3 >2.45 ng/mL (HR = 2.847, 95%CI: 1.682–4.819, P < 0.001), GPX4 <12.3 ng/mL (HR = 2.234, 95%CI: 1.458–3.425, P < 0.001), HMGB1 >15.6 ng/mL (HR = 1.856, 95%CI: 1.142–3.015, P = 0.013), and pSOFA score >8 (HR = 1.672, 95%CI: 1.089–2.567, P = 0.019) (Table 5). Bootstrap internal validation (1,000 iterations) yielded an optimism-corrected C-index of 0.794 (95%CI: 0.741–0.847), representing a modest optimism of 0.027 relative to the apparent estimate. The bootstrap-corrected AUC for the combined biomarker panel was 0.871 (95%CI: 0.826–0.916). These corrected estimates, while slightly attenuated, remain substantially superior to pSOFA score alone and preserve the primary conclusions of this study.

**TABLE 5:** Multivariate cox regression analysis of independent risk factors for 28-day mortality in pediatric sepsis patients.

Variable	β	SE	Wald χ^2^	HR	95%CI	P value
NLRP3 >2.45 ng/mL (yes vs. no)	1.046	0.269	15.123	**2.847**	1.682–4.819	**<0.001**
GPX4 <12.3 ng/mL (yes vs. no)	0.803	0.217	13.687	**2.234**	1.458–3.425	**<0.001**
HMGB1 >15.6 ng/mL (yes vs. no)	0.618	0.248	6.201	1.856	1.142–3.015	0.013
pSOFA score >8 (yes vs. no)	0.514	0.219	5.512	1.672	1.089–2.567	0.019

HR > 1 indicates an increased risk of 28-day mortality. Overall model χ^2^ = 78.456, P < 0.001; C-index = 0.821 (95%CI: 0.768–0.874); bootstrap-corrected C-index = 0.794 (95%CI: 0.741–0.847). pSOFA: pediatric sequential organ failure assessment; NLRP3: NOD-like receptor thermal protein domain-associated protein 3; GPX4: glutathione peroxidase 4; HMGB1: high mobility group box 1. The proposed biomarker cutoffs are derived from a single-center cohort and should be treated as exploratory thresholds pending external multicenter validation. Bold values indicate statistically significant associations (*P* < 0.05).

## 4 Discussion

This study presents the first comprehensive multi-pathway PCD biomarker profiling in a well-characterized pediatric PICU cohort with sepsis. By simultaneously assessing biomarkers spanning apoptosis, pyroptosis, necroptosis, ferroptosis, and DAMPs at PICU admission, we demonstrate that multiple cell death pathways are concurrently activated in pediatric sepsis and that their degree of activation correlates strongly with disease severity and 28-day mortality. Multivariate Cox regression identified four independent prognostic factors: elevated NLRP3 (pyroptosis), reduced GPX4 (ferroptosis), elevated HMGB1 (DAMP release), and pSOFA score >8. The combined PCD biomarker panel (AUC = 0.896) substantially outperformed pSOFA score alone (AUC = 0.786), highlighting the additive prognostic value of molecular cell death profiling in pediatric sepsis risk stratification.

### 4.1 NLRP3-driven pyroptosis as a central driver of pediatric sepsis mortality

The identification of NLRP3 as the strongest independent predictor of 28-day mortality (HR = 2.847) is consistent with a growing body of mechanistic and clinical evidence implicating pyroptosis as a central driver of sepsis pathology (Coll et al., 2015; Wang et al., 2025). NLRP3 inflammasome activation in macrophages, neutrophils, and endothelial cells leads to caspase-1-mediated cleavage of GSDMD, generating pore-forming N-terminal GSDMD fragments that cause osmotic cell lysis, releasing the potent pro-inflammatory cytokines IL-1β and IL-18 into the circulation (Broz et al., 2020). The downstream consequences include endothelial barrier disruption, enhanced neutrophil infiltration, and cytokine storm amplification—hallmarks of severe pediatric sepsis pathophysiology. Notably, the developing immune system of children exhibits unique inflammasome regulatory characteristics compared to adults: neonates and young children tend to display a hyperinflammatory Th2-biased response pattern with enhanced IL-18 sensitivity, potentially explaining why pyroptotic activation may be particularly impactful in pediatric populations.

### 4.2 Ferroptosis and GPX4 depletion in pediatric organ dysfunction

The robust predictive value of reduced plasma GPX4 for 28-day mortality (HR = 2.234) provides compelling clinical evidence for the pathophysiological significance of ferroptosis in pediatric sepsis. GPX4 is the master regulator of ferroptosis, responsible for reducing toxic phospholipid hydroperoxides and preventing the oxidative cascade that ultimately drives membrane rupture (Yang and Stockwell, 2016; Jiang et al., 2025). In conditions of oxidative stress and iron overload—both common in sepsis—GPX4 activity is severely compromised, unleashing unchecked lipid peroxidation in highly metabolically active cells such as renal tubular epithelium, hepatocytes, and cardiomyocytes (Chen et al., 2021). The co-elevation of MDA, 4-HNE, and ferritin in non-survivors corroborates ferroptotic pathway activation. From a developmental perspective, children have higher metabolic rates and proportionally greater oxidative stress susceptibility than adults, potentially making pediatric tissues particularly vulnerable to ferroptotic injury during sepsis. Preclinical studies demonstrating that ferroptosis inhibitors (ferrostatin-1, liproxstatin-1) attenuate organ injury in sepsis models highlight ferroptosis as an actionable therapeutic target (Tang et al., 2021).

### 4.3 DAMPs as perpetuators of sterile inflammation

The independent association of HMGB1 with 28-day mortality (HR = 1.856) reinforces the central role of DAMPs in propagating late-phase inflammation in pediatric sepsis. HMGB1, initially released from activated macrophages in response to cytokine stimulation and later passively released from necrotic and pyroptotic cells, sustains inflammation even after pathogen clearance. As a late mediator of sepsis lethality, HMGB1 binds to TLR2, TLR4, and RAGE, activating downstream NF-κB signaling and promoting transcription of additional pro-inflammatory mediators (Yang et al., 2005). The elevation of HMGB1 in non-survivors at PICU admission suggests that DAMP-mediated sterile inflammation is already pronounced early in pediatric sepsis and may represent a key determinant of subsequent mortality. Importantly, the developing immune system of infants and young children has quantitatively different HMGB1 sensitivity compared to adult immune cells, with RAGE expression patterns varying substantially across developmental stages, suggesting age-stratified biomarker interpretation may be warranted in future studies (Lotze and Tracey, 2005). Therapeutic strategies targeting HMGB1 — including anti-HMGB1 antibodies, small molecule inhibitors (e.g., glycyrrhizin), and ethyl pyruvate—have shown promising results in preclinical sepsis models. Notably, HMGB1 is a well-characterized late-phase sepsis mediator, with peak circulating levels in experimental models typically occurring 16–32 h after inflammatory stimulation—a kinetic profile distinct from early-phase markers such as NLRP3. The comparatively lower HR for HMGB1 (1.856) relative to NLRP3 (2.847) at PICU admission may therefore partly reflect measurement timing bias rather than a genuine difference in pathophysiological importance; serial sampling designs are needed to fully characterize HMGB1 prognostic kinetics. Furthermore, a sensitivity analysis excluding postoperative admissions demonstrated that the HMGB1 association was attenuated and no longer reached statistical significance (HR = 1.432, P = 0.124), consistent with surgical DAMP burden partially inflating the admission HMGB1 signal in this subgroup.

### 4.4 Crosstalk among PCD pathways: implications for pediatric immune dysregulation

Our findings provide important insights into the crosstalk between different PCD pathways in pediatric sepsis. The concurrent elevation of apoptosis, pyroptosis, necroptosis, and ferroptosis markers in all non-survivors, compared to more selective activation in survivors, suggests that simultaneous engagement of multiple PCD modalities—rather than activation of any single pathway—may be the key determinant of fatal outcomes. Several mechanisms underlie this coordinated activation: DAMPs released from pyroptotic cells trigger NLRP3 inflammasome activation in neighboring cells, perpetuating pyroptosis; oxidative stress associated with ferroptosis depletes glutathione and compromises mitochondrial membrane potential, triggering intrinsic apoptosis; and RIPK1/RIPK3-mediated necroptosis can be triggered by death receptor signals also capable of activating apoptosis, with pathway switching determined by caspase-8 availability (Newton and Manning, 2016). In the pediatric immune system, this multi-pathway convergence may be exacerbated by age-related differences in the relative abundance of caspase-8 vs. RIPK3 (determining apoptosis vs. necroptosis predominance) and by the relatively higher tissue iron stores and lower antioxidant enzyme capacity in young children (predisposing to ferroptosis).

### 4.5 Metabolic reprogramming and cell death pathway activation

Our data demonstrate that markers of ferroptosis (impaired GPX4 activity and enhanced lipid peroxidation) correlate strongly with lactate elevation and organ dysfunction, consistent with the emerging concept that metabolic reprogramming in sepsis—including mitochondrial dysfunction, Warburg-like shifts to aerobic glycolysis, and impaired fatty acid β-oxidation—creates a cellular environment uniquely susceptible to ferroptotic cell death. Mitochondrial dysfunction reduces the NADPH supply required for GPX4 activity (as GPX4 requires glutathione reduction by glutathione reductase, which itself is NADPH-dependent), establishing a direct mechanistic link between energy metabolism impairment and ferroptosis vulnerability. Similarly, NLRP3 inflammasome activation is tightly coupled to mitochondrial reactive oxygen species (mtROS) generation and impaired oxidative phosphorylation, with mtROS serving as second messengers that trigger NLRP3 oligomerization and caspase-1 recruitment (Sun et al., 2019). This bidirectional relationship between metabolic dysfunction and PCD pathway activation may constitute a vicious cycle driving progressive organ failure in pediatric sepsis. Interventions targeting mitochondrial function—including CoQ10 supplementation, mitochondria-targeted antioxidants (MitoQ), and metabolic resuscitation—may interrupt this cycle and merit investigation in pediatric clinical trials.

### 4.6 Anesthetic agents as modulators of programmed cell death: Implications for perioperative management

[Note: All observations in this section regarding anesthetic agent effects on outcomes represent hypothesis-generating secondary analyses only, and should not be interpreted as evidence of clinical efficacy. Sedative agent data are limited to binary use/non-use without dosing, duration, or timing; the reported trends are susceptible to illness-severity confounding. The cellular and molecular findings of this study carry specific implications for pediatric anesthesiology practice. Given that approximately 38.5% of PICU patients with sepsis in our cohort were postoperative cases, and that PICU management routinely involves analgosedation with agents that intersect with inflammatory signaling, the anesthesiological dimensions of PCD biology warrant systematic consideration.

Dexmedetomidine (DEX), a highly selective α2-adrenoceptor agonist widely used for cooperative sedation in the PICU and during pediatric anesthesia, has demonstrated consistent suppression of NLRP3 inflammasome activation and pyroptotic signaling in multiple experimental sepsis models. Mechanistically, DEX activates the α2-adrenoceptor/PI3K/Akt signaling axis, phosphorylating and inactivating NLRP3 protein complexes, reducing caspase-1 autoproteolytic cleavage, and diminishing GSDMD pore formation, thereby attenuating IL-1β and IL-18 release (Li et al., 2015). In animal models of lipopolysaccharide-induced sepsis, DEX pretreatment significantly reduced plasma NLRP3 and IL-18 levels and improved survival—effects abrogated by α2-adrenoceptor antagonism, confirming receptor-mediated pyroptosis suppression (Zhou et al., 2019). In our cohort, dexmedetomidine use trended toward higher prevalence in the survival group (59.5% vs. 46.7%, P = 0.085), a signal that warrants prospective evaluation. Given that NLRP3 was the strongest independent predictor of 28-day mortality (HR = 2.847), DEX-based sedation protocols in septic pediatric patients may confer organ-protective benefits through pyroptosis pathway suppression, beyond conventional sedative effects.

Propofol, the most widely administered intravenous anesthetic in pediatric practice, possesses unique antioxidant properties attributable to its 2,6-diisopropylphenol chemical structure, which resembles α-tocopherol (vitamin E) and can quench free radicals and inhibit lipid peroxidation. In the context of ferroptosis biology, propofol’s antioxidant activity may attenuate phospholipid hydroperoxide accumulation and partially compensate for GPX4 deficiency. Experimental evidence demonstrates that propofol treatment reduces MDA and 4-HNE generation, preserves mitochondrial membrane integrity in oxidative stress models, and activates Nrf2/HO-1 antioxidant pathways that can upregulate GPX4 expression (Li et al., 2022). Given that GPX4 reduction was the second strongest independent predictor of 28-day mortality (HR = 2.234) and accompanied by significantly elevated MDA and 4-HNE in non-survivors, optimized propofol-based anesthetic regimens may provide pharmacologically grounded ferroptosis-attenuating benefits in perioperative septic children. Similarly, propofol was used more frequently in surviving patients in our cohort (43.2% vs. 30.0%, P = 0.075), warranting confirmatory prospective investigation.

Midazolam, a short-acting benzodiazepine and the most commonly used sedative agent in the PICU after dexmedetomidine and propofol, acts primarily through GABA-A receptor potentiation. Emerging preclinical evidence suggests that benzodiazepines may modulate neuroinflammatory pathways indirectly by suppressing microglial NLRP3 activation and reducing central nervous system IL-1β release, though clinical data in sepsis remain limited. In our cohort, midazolam use data are reported in Table 1 for completeness. Given the limited mechanistic evidence and the current study’s retrospective design, dedicated investigation of midazolam’s effects on PCD pathways in pediatric sepsis is warranted. Ketamine has also been reported to exert immunomodulatory effects on peripheral inflammatory responses, although its relationship with PCD signaling in pediatric sepsis remains unclear (De Kock et al., 2013).

While volatile anesthetics such as sevoflurane have demonstrated organ-protective effects relevant to PCD pathway modulation in animal models, their application in PICU settings requires specialized delivery equipment not routinely available; their clinical relevance to ongoing PICU sedation is therefore limited and warrants separate investigation.

### 4.7 Therapeutic opportunities and future directions

The mechanistic insights generated by this study identify several promising therapeutic targets for pediatric sepsis, extending beyond conventional antimicrobial and organ support strategies. For pyroptosis, NLRP3 inflammasome inhibitors—including the clinically tested small molecule MCC950, the FDA-approved gout drug colchicine (which indirectly disrupts inflammasome assembly), and novel covalent NLRP3 inhibitors—represent attractive candidates for adjunctive therapy in pediatric sepsis with elevated NLRP3 (Coll et al., 2015). For ferroptosis, clinical-stage interventions include ferrostatin-1 analogues, Nrf2 activators (sulforaphane, bardoxolone), and iron chelation strategies (Tang et al., 2021). HMGB1-targeting approaches, including humanized anti-HMGB1 antibodies, glycyrrhizin, and ethyl pyruvate, have shown promising preclinical results and require pediatric clinical validation. Importantly, given the multi-pathway nature of PCD activation observed in this study, combination strategies targeting two or more PCD pathways simultaneously—particularly combined pyroptosis/ferroptosis inhibition—may be required to meaningfully interrupt the cell death cascade in the most critically ill pediatric patients. Future research should prioritize pediatric pharmacokinetic studies of PCD-targeting agents, given developmental differences in drug metabolism and biomarker thresholds across age groups.

### 4.8 Limitations

This study has several limitations. First, the retrospective single-center design introduces potential selection bias and limits generalizability. Second, PCD biomarker measurements were performed at a single time point (PICU admission), and temporal dynamics during sepsis progression were not captured. Third, while plasma biomarker levels are clinically accessible, they may not fully reflect tissue-level PCD activation or cellular sources of circulating biomarkers. Fourth, the effect of pre-PICU interventions—including pre-hospital or emergency department treatment—on biomarker levels could not be fully controlled. Fifth, given that multiple comparisons were performed across many biomarkers, some false-positive associations cannot be excluded, though consistent patterns within each PCD pathway reduce this concern. Sixth, the observational association between anesthetic agent use and outcome (dexmedetomidine, propofol) requires validation in prospective randomized trials. Future multicenter prospective pediatric studies with serial biomarker measurements, tissue-level validation, age-stratified analysis, and mechanistic evaluation of anesthetic agent effects on PCD pathway activation are needed to validate and extend these findings.

## 5 Conclusion

This study demonstrates that multiple programmed cell death pathways—apoptosis, pyroptosis, necroptosis, and ferroptosis—are simultaneously activated in pediatric sepsis, with the degree of concurrent pathway engagement strongly associated with organ dysfunction severity and 28-day all-cause mortality. Pyroptosis (as reflected by NLRP3 elevation) and ferroptosis (as reflected by GPX4 reduction) are the most potent independent predictors of poor prognosis, while HMGB1 reflects DAMP-driven perpetuation of sterile inflammation. The combined PCD biomarker panel provides significant additive prognostic value beyond pSOFA score alone and identifies mechanistically distinct therapeutic targets. From a pediatric anesthesiology perspective, these findings provide a compelling mechanistic rationale for investigating dexmedetomidine and propofol as adjunctive anesthesia-based organ-protective strategies targeting specific PCD pathways in critically ill septic children. These findings advance our understanding of the molecular mechanisms underlying pediatric sepsis-associated organ failure and establish the clinical prognostic utility of multi-pathway PCD biomarker profiling in the PICU setting.

## Data Availability

The datasets analyzed in this study are not publicly available because they contain potentially identifiable patient information. Requests to access the datasets should be directed to the corresponding author, Xifeng Zhang (Xifeng86928@163.com), and will be considered in accordance with institutional and ethical requirements.
